# Correction: Giving birth: A hermeneutic study of the expectations and experiences of healthy primigravid women in Switzerland

**DOI:** 10.1371/journal.pone.0314064

**Published:** 2024-11-13

**Authors:** Valerie Fleming, Franziska Frank, Yvonne Meyer, Jessica Pehlke-Milde, Piroska Zsindely, Harriet Thorn-Cole, Claire de Labrusse

The affiliation for the seventh author is incorrect. The correct affiliation is not indicated. Claire de Labrusse is not affiliated with #3 but with: HESAV School of Health Sciences, HES-SO University of Applied Sciences and Arts Western Switzerland.
