# Supplementary material for: Giving birth: A hermeneutic study of the expectations and experiences of healthy primigravid women in Switzerland
Source: PLoS One. 2022 Feb 4;17(2):e0261902. doi: 10.1371/journal.pone.0261902 (PMC8815900; doi:10.1371/journal.pone.0261902)
Supplement: S1 File — (DOCX) [file pone.0261902.s001.docx]

**Interview Guide – Key questions**

**Interview 1:**

- “What are your expectations of birth?”

From this interview and all the others, sub-questions will be generated from the initial response of each participant. Field notes will be used to complete each oral interview. The other main questions are likely to be:

- “Who or what influenced these expectations?”
- “What options do you see available to you?”

**Interview 2 :**

- “What are your expectations of birth now?”
- “What preparations are you making for the birth OR How are you preparing for the birth
- “Have you a written birth plan?”

**Interview 3** :

- “How was your birth in relation to your earlier expectations?”
- “How was it for you?”

If changed:

- “How was it different”
- “Who or what had the most influence on how you gave birth? "

**Interview 4** :

- “How do you feel about your birth experience?”
- “Would you have wanted something different?”
- “What would you consider for a future birth?”
