# Supplementary material for: Giving birth: A hermeneutic study of the expectations and experiences of healthy primigravid women in Switzerland
Source: PLoS One. 2022 Feb 4;17(2):e0261902. doi: 10.1371/journal.pone.0261902 (PMC8815900; doi:10.1371/journal.pone.0261902)
Supplement: S2 File — (DOCX) [file pone.0261902.s002.docx]

**Guide d’entretient – Questions clés**

Entretient 1: “Quelles sont vos attentes par rapport à l’accouchement ? »

Depuis cet entretient et tous les autres, des sous-questions seront générées depuis la réponse initiale de chaque participante. Des notes de terrain seront utilisées pour compléter chaque entretient oral.

Les autres questions principales sont susceptibles d'être: Qui ou quoi a influencé ces attentes? Quelles sont les options que vous voyez à votre disposition?

Entretient 2: «Quelles sont vos attentes aujourd'hui par rapport à l’accouchement?" "Quels cours de préparation à la naissance fréquentez-vous ?" "Avez-vous écrit un plan de naissance?"

Entretient 3: «Quelle a été votre expérience d’accouchement?" "Comment était-ce pour vous" (Si modifications) "Comment était-ce diffèrent" "Qui ou quoi a exercé le plus d'influence sur la façon dont vous avez accouché?"

Entretient 4: «Comment vous sentez-vous maintenant par rapport à ​​votre expérience de l'accouchement?" "Auriez-vous voulu quelque chose de différent ?" "Qu'est-ce que vous considériez maintenant pour une autre naissance?".
