# Supplementary material for: Giving birth: A hermeneutic study of the expectations and experiences of healthy primigravid women in Switzerland
Source: PLoS One. 2022 Feb 4;17(2):e0261902. doi: 10.1371/journal.pone.0261902 (PMC8815900; doi:10.1371/journal.pone.0261902)
Supplement: S3 File — (DOCX) [file pone.0261902.s003.docx]

Interviewfragen

Interview 1: Was erwarten Sie bezüglich Geburt?

Was sind Ihre Erwartungen bezüglich Geburt?

Welches sind Ihre Geburtserwartungen?

Ausgehend von/Basierend auf der jeweiligen Antwort der Teilnehmerinnen auf diese initiale Fragen werden in diesem sowie in allen folgenden/anderen Interviews Unterfragen gebildet/formuliert. Zudem werden Feldnotizen angefertigt, um die verbale Konversation/das verbale Gespräch zu ergänzen.

Andere/weitere Kernfragen/Leitfragen werden voraussichtlich sein/wie folgt lauten: Wer oder was hat diese Erwartungen beeinflusst? Welche vorhandenen Möglichkeiten sehen Sie für sich?

Interview 2: Was erwarten Sie jetzt bezüglich Geburt?

Was sind jetzt Ihre Erwartungen bezüglich Geburt?

Welches sind jetzt Ihre Geburtserwartungen?

Was machen Sie bezüglich Geburtsvorbereitung?

Haben Sie Ihre Wünsche bezüglich Geburt niedergeschrieben?

Interview 3: Wie haben Sie die Geburt erlebt/erfahren?

Wie war das für Sie? (falls das Erleben/die Erfahrung nicht mit den Erwartungen übereinstimmt):

Wie haben sie sich unterschieden?

Wo lagen die Unterschiede? Wo sind sie voneinander abgewichen?

Wer oder was hat den grössten Einfluss darauf ausgeübt, wie Sie geboren haben?

Interview 4: Wie fühlen Sie sich jetzt bezüglich Ihrer Geburtserfahrung?

Gibt es etwas, das Sie sich rückblickend gerne anders gewünscht hätten/das Sie gerne anders gehabt hätten?

Was würden Sie jetzt für eine nächste Geburt beachten/berücksichtigen?
