# Supplementary material for: Giving birth: A hermeneutic study of the expectations and experiences of healthy primigravid women in Switzerland
Source: PLoS One. 2022 Feb 4;17(2):e0261902. doi: 10.1371/journal.pone.0261902 (PMC8815900; doi:10.1371/journal.pone.0261902)
Supplement: S4 File — (DOCX) [file pone.0261902.s004.docx]

**Guida d’intervista - Domande chiavi**

*Intervista 1*: “Quali sono le Sue aspettative riguardo al parto ?”

Altre domande saranno generate in risposta a questa domanda. Ogni intervista sarà completata con note della ricercatrice.

Le altre domande potranno essere: “Cosa o chi ha influenzato le Sue aspettative ?”, “Quali sono le opzioni a Sua disposizione secondo Lei ?”

*Intervista 2*: “Quali sono oggi le Sue aspettative riguardo al parto ?”, “Quali lezioni di preparazione alla nascita segue ?”, “Lei ha scritto un piano di nascita ?”

*Intervista 3*: “Come ha vissuto l’esperienza del parto ?”, “Com’è andata per Lei ?”, “Se modifica, cos’è cambiato?”, “Cosa o chi ha influenzato di più il modo in cui ha partorito?”

*Intervista 4*: “Come si sente oggi quando pensa alla Sua esperienze di parto?”, “Lei avrebbe voluto che si sia svolto in un altro modo?”, “Lei cosa pensa o che aspettative ha per una seconda nascita?”
