# Supplementary material for: Giving birth: A hermeneutic study of the expectations and experiences of healthy primigravid women in Switzerland
Source: PLoS One. 2022 Feb 4;17(2):e0261902. doi: 10.1371/journal.pone.0261902 (PMC8815900; doi:10.1371/journal.pone.0261902)
Supplement: Attachment — Submitted filename: Rebuttal lettre.docx [file pone.0261902.s005.docx]

Lieu, November 2022

Dear Dr. David Desseauve,

We would like to thank you for considering our manuscript, and the reviewers for their time and comments. We have edited our manuscript and supporting information to address the raised concerns:

| Editor’s and reviewers’ comments | Response and/or amendments |
| --- | --- |
| 1. Please ensure that your manuscript meets PLOS ONE's style requirements, including those for file naming. | 1. The style of the manuscript has been changed to meet the PLOS One requirements. |
| 2. Please review your reference list to ensure that it is complete and correct. If you have cited papers that have been retracted, please include the rationale for doing so in the manuscript text, or remove these references and replace them with relevant current references. Any changes to the reference list should be mentioned in the rebuttal letter that accompanies your revised manuscript. If you need to cite a retracted article, indicate the article’s retracted status in the References list and also include a citation and full reference for the retraction notice. | 2. The following references have been added following the comments of reviewer 2:   - 11: Morton R, Burton AE, Kumar P, Hyett JA, Phipps H, McGeechan K, et al. Cesarean delivery: Trend in indications over three decades within a major city hospital network. Acta Obstetricia et Gynecologica Scandinavica. 2020;99: 909–916. doi:10.1111/aogs.13816 - 13: Sobhy S, Arroyo-Manzano D, Murugesu N, Karthikeyan G, Kumar V, Kaur I, et al. Maternal and perinatal mortality and complications associated with caesarean section in low-income and middle-income countries: a systematic review and meta-analysis. The Lancet. 2019;393: 1973–1982. doi:10.1016/S0140-6736(18)32386-9 |
| 3. Please include a copy of the interview guide used in the study, in both the original language and English, as Supporting Information, or include a citation if it has been published previously | 3. The interview guides in English, French, German and Italian are now included. |
| 4. Please provide additional details regarding participant consent. In the ethics statement in the Methods and online submission information, please ensure that you have specified what type you obtained (for instance, written or verbal, and if verbal, how it was documented and witnessed). If your study included minors, state whether you obtained consent from parents or guardians. If the need for consent was waived by the ethics committee, please include this information. | 4. We have specified in our manuscript that women were asked to sign a consent form. |
| 5. We note that the grant information you provided in the ‘Funding Information’ and ‘Financial Disclosure’ sections do not match. When you resubmit, please ensure that you provide the correct grant numbers for the awards you received for your study in the ‘Funding Information’ section. | 5. When submitting, we will ensure the funding information matches between the manuscript and the financial disclosure. |
| 6. Thank you for stating the following financial disclosure:  "The funders had no role in study design, data collection and analysis, decision to publish, or preparation of the manuscript."  At this time, please address the following queries:  a) Please clarify the sources of funding (financial or material support) for your study. List the grants or organizations that supported your study, including funding received from your institution.  b) State what role the funders took in the study. If the funders had no role in your study, please state: “The funders had no role in study design, data collection and analysis, decision to publish, or preparation of the manuscript.” c) If any authors received a salary from any of your funders, please state which authors and which funders. d) If you did not receive any funding for this study, please state: “The authors received no specific funding for this work.”  Please include your amended statements within your cover letter; we will change the online submission form on your behalf. | 6. We have stated that the funders had no role in study design, data collection and analysis, decision to publish or preparation of the manuscript. |
| 7. Please note that in order to use the direct billing option the corresponding author must be affiliated with the chosen institute. Please either amend your manuscript to change the affiliation or corresponding author, or email us at [plosone@plos.org](mailto:plosone@plos.org) with a request to remove this option. | 7. Please, would you mind changing the corresponding author to Valerie Fleming of the Liverpool John Moores University. |
| 8. PLOS requires an ORCID iD for the corresponding author in Editorial Manager on papers submitted after December 6th, 2016. Please ensure that you have an ORCID iD and that it is validated in Editorial Manager. | 8. Prof. Valerie Fleming’s ORCID number is **0000-0002-4672-4843** |
| 9. Please upload a new copy of Figure 1 as the detail is not clear. " | 9. Figure 1 quality has been improved and is uploaded |
| 10. Reviewer #1: I suggest to remove l152:"three gave Birth" wich is not usefull and suggests possible other interviewers biais (ethnicity, gender, nationality, age …).  Fig 1 appearence should be improve. | We would like to thank reviewer 1's positive and constructive feedback. We agree with the made comments and removed l172: "three gave birth", and as above (see item 9) improved figure 1. |
| 11. Reviewer #2:  perhaps include references to concerns as to the indication recorded as a justification for the CS i.e. if its fetal hypoxia is this corroborated in the notes. This was highlighted re maternal request in Brazil. Suggest also adding to concerns re the consequences of rising CS rates in terms of placenta accreta/percreta/increta and also the rising CS rates ineh USA accompanied by an increase in maternal deaths.... | 11.1: L66-67: a sentence was added following the suggestions made by reviewer 2.  11.2 : L.70 : another sentence was added following these suggestions reporting the concerns re the rising CS rates.  2 References were then added. See point 1. |

We would like to thank you for giving us the opportunity to enhance our manuscript with your constructive comments. We have made sure that we have incorporated your feedback and hope that our manuscript now meets the PLOSone standards and convinces you to accept it for publication. We look forward to hearing from you regarding our submission. We would be happy to answer any further queries and comments that you may have.

Claire de Labrusse, on behalf of Valerie Fleming
